# Investigating the efficacy of vacuum sealing drainage versus traditional negative pressure drainage in treating deep incision infections following posterior cervical internal fixation—a retrospective cohort study

**DOI:** 10.1186/s40001-024-01717-7

**Published:** 2024-02-15

**Authors:** Jianhua Li, Dawei Jiang, Zhengqi Chang

**Affiliations:** Department of Orthopaedic Surgery, 960th Hospital of PLA, Shifan road, Tianqiao district, Jinan, 250031 China

**Keywords:** Cervical spondylosis, Deep incision infections(DII), Posterior cervical internal fixation, Vacuum sealing drainage (VSD), Traditional negative pressure drainage (TND)

## Abstract

**Background:**

Assessing the efficacy and safety of Vacuum Sealing Drainage (VSD) in treating deep incision infections (DII) following posterior cervical internal fixation.

**Methods:**

We retrospectively studied the clinical effects of VSD and Traditional Negative Pressure Drainage (TND) on 12 patients with deep incision infection after posterior cervical fixation surgery who were treated in our department from 2012 to 2020. A comparison of patient-related factors (age, gender, BMI, comorbidities, initial internal fixation surgery segment, preoperative laboratory inflammation indicators) and surgical-related factors (postoperative duration of fever, positive rate of drainage fluid bacterial culture, Visual Analogue Scale (VAS) score at 3 days after surgery, laboratory indicators at 3 days after surgery, debridement frequency and drainage time, hospital stay, internal fixation retention rate, and infection recurrence rate) between the VSD group and the TND group was conducted using independent sample *t* tests to draw experimental conclusions.

**Results:**

This study included 12 patients, with six cases of VSD (5 males and 1 female) and six cases of TND (4 males and 2 females). The VSD group had significantly lower postoperative fever time (1.50 ± 0.46 days vs. 4.28 ± 0.97 days, *P* < 0.05), a higher positive rate of bacterial cultures in drainage fluid (5/6 vs. 2/6, *P* < 0.05), lower 3 day VAS scores (3.13 ± 0.83 vs. 3.44 ± 0.88, *P* < 0.05), lower 3 day CRP levels (66.89 ± 23.65 mg/L vs. 57.11 ± 18.18 mg/L, *P* < 0.05), a shorter total drainage time (14.50 ± 2.98 days vs. 22.56 ± 3.01 days, *P* < 0.05), and a higher total drainage flow rate (395.63 ± 60.97 ml vs. 155.56 ± 32.54 ml, *P* < 0.05) than the TND group (the total drainage volume throughout the entire treatment process). In addition, the frequency of debridement (2.67 ± 0.52 times vs. 3.17 ± 0.41 times, *P* < 0.05) and average hospital stay (23.13 ± 3.27 days vs. 34.33 ± 6.86 days, *P* < 0.05) were significantly lower in the VSD group, although both groups retained internal fixation.

**Conclusions:**

VSD is a secure and effective treatment for deep incision infections that results from cervical posterior internal fixation surgery.

## Background

In the past few years, as cervical spondylosis has become more common, the use of posterior cervical surgery and internal fixation has risen in tandem [1]. Following posterior spinal internal fixation surgery, postoperative surgical site infections (SSIs) and inadequate incision healing are the two most common complications, with an overall incidence rate of 2–4.15% [2, 3]; specifically, in the cervical segments, it is approximately 3%. After spinal surgery, infections can lead to internal fixation failure and a bad clinical outcome. Not only do they prolong hospitalization, but they also increase the patient's pain. In some cases, they may require revision surgery, elevating the risk of surgery and potentially causing spinal deformities. Spinal infections can be caused by various patient-related factors such as malignancy, obesity, existing diseases, taking immunosuppressant medications, following doctor's orders, and multiple accidental injuries. In addition, surgical factors such as the time of the surgery, the position and number of internal fixation segments can also contribute to spinal infections [4–6].

The cervical spine's particular anatomical location and structure make treating deep incision infections (DII) after a posterior cervical fixation surgery critical to avert a bad prognosis caused by infection dissemination. In addition to basic antibiotic treatment, conventional treatments mainly consist of lesion debridement (some patients necessitate the removal of internal fixation devices), repeated dressing changes, and lesion flushing and drainage surgery [7–10]. Debridement of lesions is a must; however, the cervical spine's unique anatomy makes it difficult to completely debride. The bacterial biofilm on the surface of the internal fixation device is a major cause of the ineffectiveness of antibiotics and the recurrence of infection, thus, the removal of the internal fixation device is sometimes the only solution, though it can lead to pain and neurological symptoms. By using irrigation and drainage, the success rate of spinal infection treatment and the rate of internal fixation devices remaining in place was improved; however, slow wound healing prolonged the hospital stay, negatively impacting the quality of life of patients during treatment [11], as well as increasing the risk of infection due to poor drainage. In addition, local pain and neurological symptoms were also observed [10, 12].

VSD is a popular technique for treating limb trauma and infection, demonstrating positive effects in the clinical management of bone and soft-tissue infections in the limbs. The mechanisms of action include reducing bacterial levels, removing inflammatory components, minimizing swelling, increasing blood flow to the tissues, aiding vascular regeneration, and stimulating the formation of granulation tissue [13]. Although there have been reports of VSD being used to treat superficial infections after spinal surgery, there is still limited evidence of its use to address DIIs following cervical posterior fixation surgery. To assess the efficacy and safety of VSD in treating DII following postcervical internal fixation, we compared the clinical data of the VSD and TND groups and summarized the results.

## Methods

### Patient population

This work has been reported in line with the STROCSS criteria. The definition of deep infection used in this study was the formation of abscesses or other infectious manifestations in the deep soft tissues, muscles, and fascia [14]. A comprehensive evaluation of deep infection after spinal surgery was conducted by examining clinical manifestations (e.g., incision dehiscence, redness and swelling, pain, fever, and pus discharge), abnormal elevation of laboratory inflammatory markers, and imaging findings suggestive of infection. Patients with skin and subcutaneous tissue infection and delayed healing were excluded from the study. After a thorough screening and evaluation of the medical records of all patients who underwent posterior cervical fixation surgery, the study subjects were selected for this study. This research included 12 patients who had undergone posterior cervical fixation operations and were diagnosed with DII. They were divided into two groups: the VSD group (5 males and 1 female, average age 51.72 ± 11.17 years) and the TND group (4 males and 2 females, average age 54.45 ± 9.79 years).

### Surgical technique

**Fig. 1:**
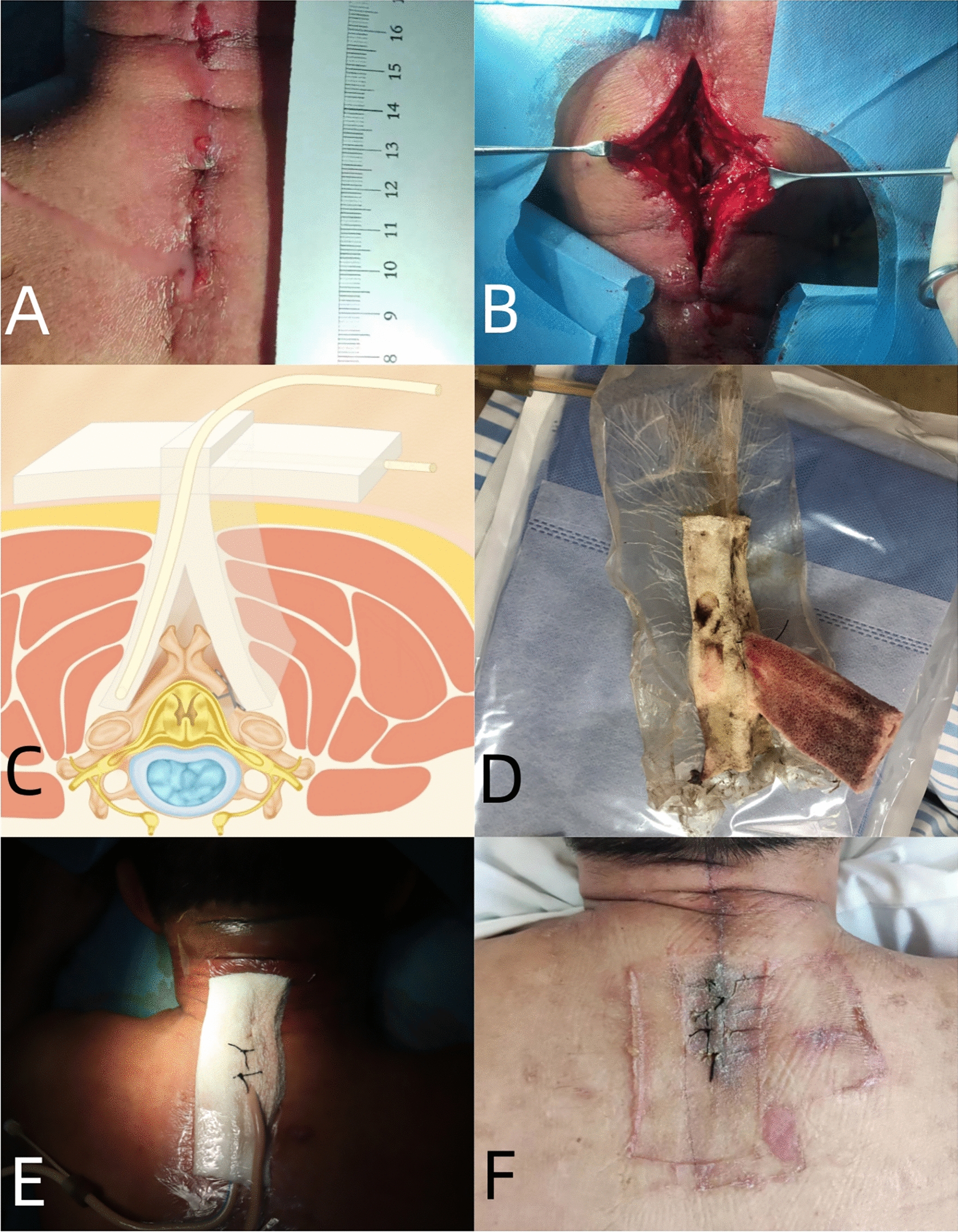
Schematic Diagram of VSD Sponge Placement. **A**, **B** A 72-year-old male patient was diagnosed with DDI and treated with debridement and VSD; **C,** **D** schematic diagram of VSD sponge placement; **E,** **F** after replacing the VSD sponge for 3 times, the symptoms disappeared, the infection was cured, and the internal fixation was retained

**Fig. 2:**
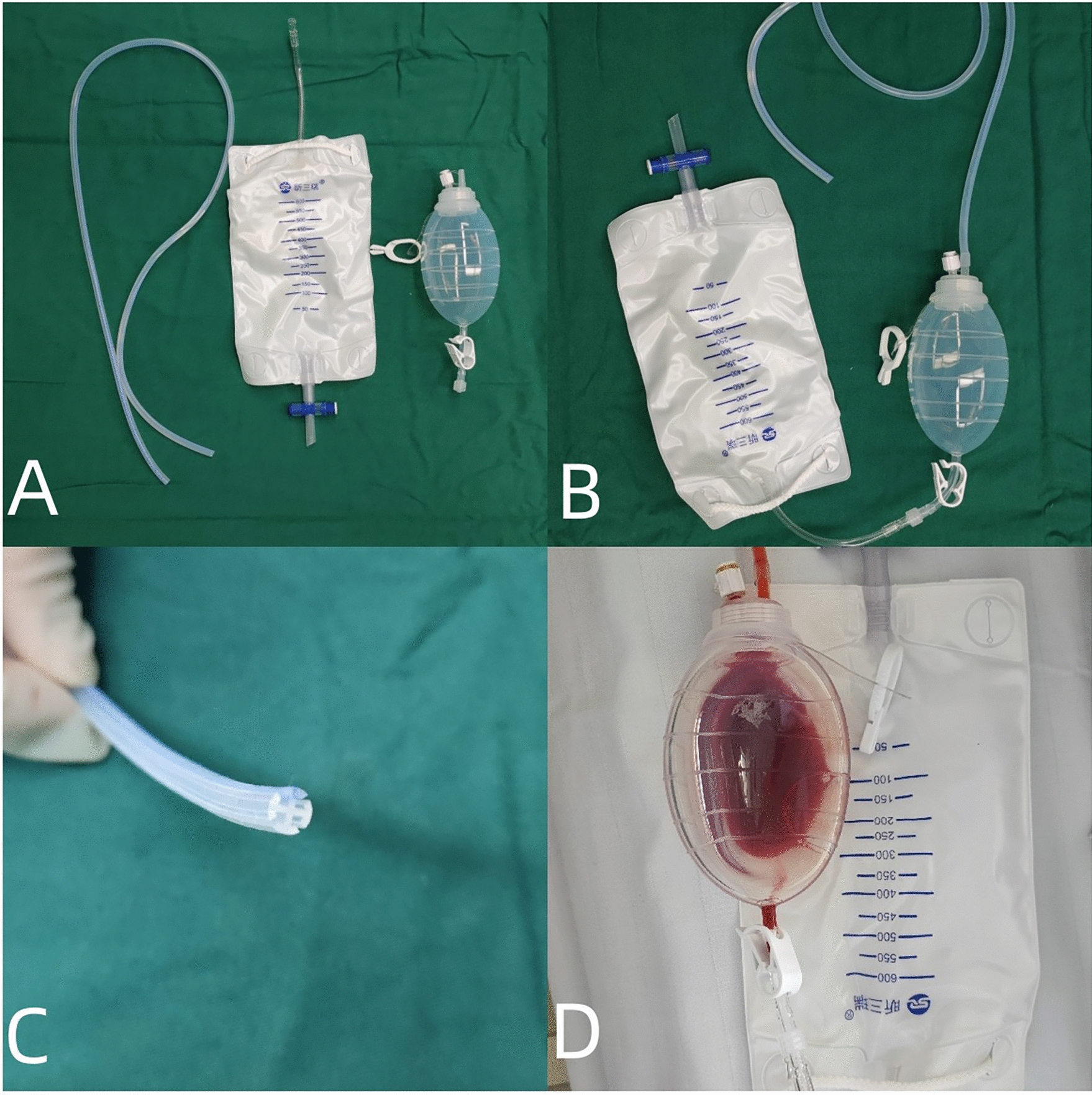
Schematic Diagram of common negative pressure drainage tube in TND group. **A,** **B** Overview of the common negative pressure drainage tubes used in the TND group is presented; **C** visual of the beginning of a siphon drainage tube; **D** appearance of the negative pressure drainage ball

**Fig. 3:**
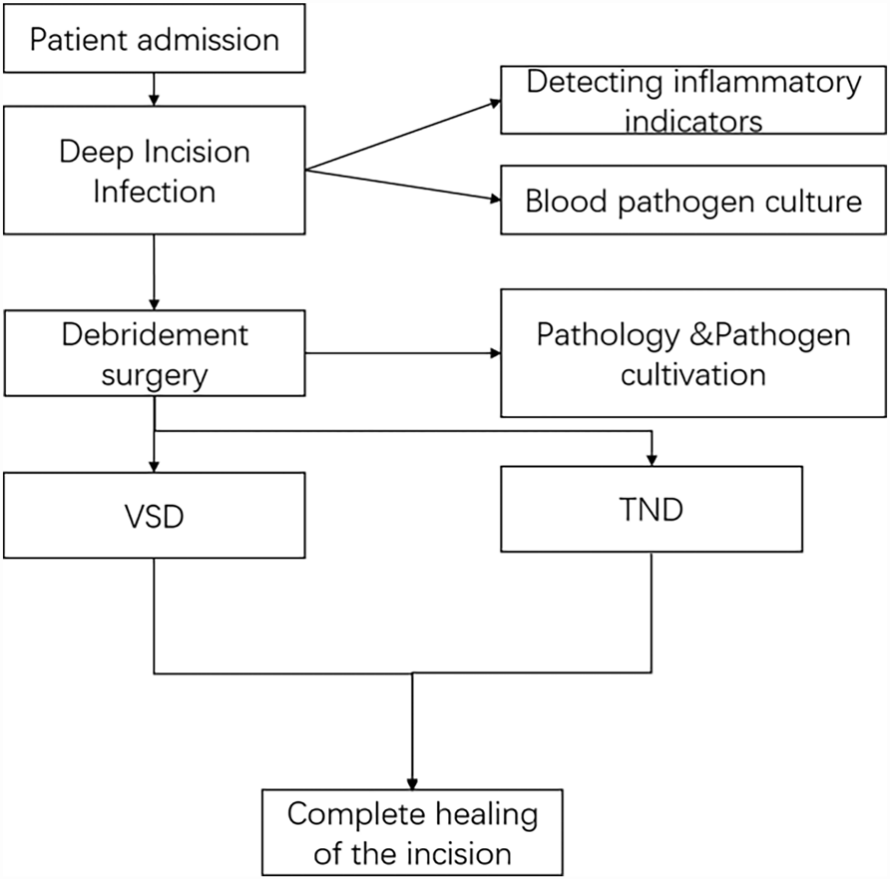
Study flow chart

All 12 patients underwent a standard debridement and irrigation procedure with local anesthesia. The infected and necrotic tissue was removed and the wounds were rinsed with chlorhexidine, hydrogen peroxide, and saline for 15 min. For the VSD group, two sponges were cut to fit the depth and length of the wound. The sponge was then sutured and fixed with silk thread to prevent it from sliding and displacing. The cavity was filled and eliminated as much as possible. The secondary sponge was placed to cover the wound edge of the skin and the primary sponge was passed through the middle. Both sponges were kept in a negative-pressure suction state (Fig. 1). The hospital bed is equipped with a negative pressure suction device, which is connected to two drainage tubes. This device is capable of setting and maintaining a constant negative pressure value between 0.03 and 0.04 MPa. For the TND group, a siphon and negative-pressure drainage ball were placed first, followed by suturing the wound with silk thread (Fig. 2). The treatment process diagrams for two groups of patients can be seen in Fig. 3.

### Post‑operative management

Patients were given antibiotics both before and after debridement surgery. The selection of antibiotics was based on the results of drug sensitivity tests, and if there were no such results, then a second-generation cephalosporin antibiotic was chosen. During the procedure, tissue samples and drainage fluid after the operation were taken for bacterial culture and drug sensitivity tests, and the use of antibiotics was adjusted accordingly. All infected patients were administered intravenous antibiotics for a minimum of 3 weeks, followed by oral sensitive antibiotic treatment for an additional 5 weeks, for a total of at least 8 weeks.

To control deep infection, VSD sponges should be replaced based on the condition of the drainage fluid. If the fluid is bacteria-free three times or more and the area around the internal fixation device and pedicle is covered with granulation tissue, it is assumed that the infection has been cured. Each time the sponge is changed, the depth and amount of the sponge should be decreased according to the condition. The incision should also be reduced until it is completely closed and the VSD device is removed. The criteria for VSD were removed, mainly focusing on the absence of redness or swelling at the incision site, the absence of any dead space in the incision, and the presence of fresh granulation tissue on the wound. The patient’s inflammatory indicators (WBC, CRP, ESR, etc.) showed a significant decrease to almost normal levels after three consecutive tests, and the total drainage volume within 24 h was ≤ 20 ml. After that, regular dressing changes should be done until the wound is completely healed. The TND group underwent debridement and drainage surgery every 7–10 days, depending on the condition of the wound. Once the total drainage volume is ≤ 20 ml within 24 h, the drainage tube can be removed. In addition, it is important to regularly change the wound dressing until the incision is fully healed.

### Data collection

Standardized data collection scales should be used to collect and record all pertinent information in patient medical records, such as patient-related indicators (e.g., age, gender, BMI, comorbidities, initial internal fixation surgery segment, and preoperative laboratory inflammation indicators) and surgical-related indicators (e.g., duration of postoperative fever, positive rate of bacterial culture in drainage fluid, VAS score at 3 days after surgery, laboratory validation indicators at 3 days after surgery, drainage placement time and frequency in both groups, hospitalization time, retention rate of internal fixation, and infection recurrence rate).

### Statistical analysis

The analysis of data was performed using SPSS 16.0, and the results were presented as mean ± standard deviation. Initially, normality and homogeneity of variance were assessed. If the data followed a normal distribution, the independent sample *t* test was used to compare the two groups. However, if the data did not exhibit a normal distribution, the rank sum test was utilized instead. For intergroup comparison of categorical data, Fisher's exact probability method was employed. A statistically significant difference was denoted by P < 0.05.

## Results

In the VSD group, there were three patients with diabetes, two with diabetes accompanied by hypertension, and one with coronary heart disease. Meanwhile, in the routine debridement and drainage group, there were four cases of diabetes, one of diabetes with hypertension, and one of hypertension with rheumatoid arthritis. This is summarized in Table 1.

**Table 1:** Comparison of preoperative general conditions between the two groups

	VSD Group	TND Group	*P*
Age	51.72 ± 11.17	54.45 ± 9.79	0.716
Gender			
Male	5	4	
Female	1	2	
BMI	24.71 ± 3.36	24.89 ± 6.97	0.569
Comorbidities	6/6	6/6	
Surgical levels	1.38 ± 0.52	1.33 ± 0.50	0.318
Length of incision (cm)	9.13 ± 1.56	9.44 ± 1.51	0.287
Laboratory examination			
WBC(× 10^9^/L)	10.13 ± 2.30	10.33 ± 2.55	0.293
ESR(mm/h)	63.75 ± 17.69	65.67 ± 19.67	0.619
CRP (mg/L)	50.13 ± 20.51	49.11 ± 19.94	0.801

The comparison of related indicators between the VSD group and the TND group is presented in Table 2. The VSD group had significantly lower postoperative fever time (1.50 ± 0.46 days vs. 4.28 ± 0.97 days, *P* < 0.05), a higher positive rate of bacterial cultures in drainage fluid (5/6 vs. 2/6, *P* < 0.05; In the VSD group, Staphylococcus aureus was found in two cases, Staphylococcus epidermidis in one case, Pseudomonas aeruginosa in one case, and Escherichia coli in one case. In the TND group, Pseudomonas aeruginosa was present in one case and Enterococcus faecalis in one case), lower 3 day VAS scores (3.13 ± 0.83 vs. 3.44 ± 0.88, *P* < 0.05), lower 3 day CRP levels (66.89 ± 23.65 vs. 57.11 ± 18.18, *P* < 0.05), a shorter total drainage time (14.50 ± 2.98 days vs. 22.56 ± 3.01 days, *P* < 0.05), and a higher total drainage flow rate (395.63 ± 60.97 ml vs. 155.56 ± 32.54 ml, *P* < 0.05) than the TND group (the total drainage volume throughout the entire treatment process). In addition, the frequency of debridement (2.67 ± 0.52 vs. 3.17 ± 0.41, *P* < 0.05) and average hospital stay (23.13 ± 3.27 vs. 34.33 ± 6.86, *P* < 0.05) were significantly lower in the VSD group. On discharge, the VAS scores for both groups were roughly the same and the internal fixation remained intact. Neither group experienced any recurrent cases or cerebrospinal fluid leakage.

**Table 2:** Comparison of postoperative infection indexes between the two groups

	VSD Group	TND Group	*P*
Implant retention rate	6/6	6/6	
Duration of fever after debridement (day)	1.50 ± 0.46	4.28 ± 0.97	0.001
Laboratory examination (3 days after surgery)			
WBC(× 10^9^/L)	7.88 ± 1.73	8.22 ± 1.99	0.571
ESR (mm/h)	47.00 ± 21.03	50.89 ± 14.26	0.401
CRP(mg/L)	66.89 ± 23.65	57.11 ± 18.18	0.668
VAS (3 days after surgery)	3.13 ± 0.83	3.44 ± 0.88	0.349
Positive microorganism results	5/6	2/6	0.001
Replacement times	2.67 ± 0.52	3.17 ± 0.41	0.032
The volume of liquid drainage fuid (ml)	395.63 ± 60.97	155.56 ± 32.54	0.001
Drainage time (day)	14.50 ± 2.98	22.56 ± 3.01	0.001
Hospitalization (day)	23.13 ± 3.27	34.33 ± 6.86	0.031
VAS (discharge)	2 ± 0.76	2.11 ± 0.60	0.479
Incidence of recurrence	0/6	0/6	

## Discussion

Post-spinal DII is a rare but serious postoperative complication, with a poor prognosis. Contributing factors include patient characteristics such as malignancy, obesity, underlying diseases, immunosuppressant drug use, non-adherence to postoperative instructions, and recurrent trauma. Surgical factors include length of operation, increased intraoperative blood loss, and extended fixation segments [3, 5, 15, 16]. Although antibiotics, adequate drainage, and proper wound care can reduce the risk of infection, the incidence of postoperative infection remains around 2–5% [2, 4, 17–19]. Conventional treatment for postoperative spinal infection involves debridement and drainage surgery along with the use of sensitive antibiotics [20]. Due to the intricate anatomy and single-approach nature of open posterior spinal internal fixation surgery, traditional debridement for infected focus can be a lengthy process [21]. Moreover, some patients may require the removal of the internal fixation device or further internal fixation revision surgery to restore spinal stability, which increases the risk of surgical trauma and mortality [22]. In addition, traditional debridement for infected focus has a high recurrence rate, making it especially dangerous for patients with pre-existing conditions and poor overall health.

Following a posterior internal fixation surgery of the cervical spine, there are three main characteristics of the DII: first, the cervical spine is a transitional area between the neck and chest, with complex motor functions, making it difficult to bind and secure the wound. Second, the conventional posterior cervical surgery involves a median incision, which lacks great vessels on either side of the incision, resulting in poor blood supply and difficulty in wound healing after infection. Finally, the surgical area is close to important organs, blood vessels, and nerves, with intricate anatomical structures and limited surgical space, making it difficult to completely debride the area after infection. Therefore, DII after cervical spine surgery not only increases the risk of infection dissemination and other serious complications, but also limits the scope of lesion debridement, which is a major contributor to the extended treatment period, high internal fixation revision rate, and high rate of infection recurrence [17, 23].

The use of VSD technology has been well-documented in the treatment of bone and soft tissue infections in the limbs. However, there are still few reports on its application in the treatment of DII after spinal surgery, particularly after posterior cervical fixation surgery. In comparison with traditional debridement and drainage surgery, VSD can not only rapidly control the spread of infection, but also significantly reduce inflammation and edema, and promote wound healing [24]. Both Canavese and Akhter's studies support this notion [11, 25]. In comparison with the TND group, the VSD group had a significantly shorter postoperative fever duration, as well as a more marked improvement in ESR and CRP levels at 3 days, VAS score at 3 days, internal fixation retention rate, and average incision healing time. These findings are in line with previous research [21, 26]. There was no significant difference in terms of surgical time and intraoperative bleeding volume. Taking into account the results reported by Kale and Labeler et al. [27], we still believe that VSD is a reliable treatment for DII after posterior cervical fixation surgery.

VSD technology's ability to treat DII may be linked to its capacity to destroy bacterial biofilm, quickly eliminate necrotic tissue, and promote vascularization [28–30]. Owing to the fact that most patients with DII have undergone long-term antibiotic treatment, the rate of bacterial culture that is positive is usually low. This is tightly connected to the bacterial biofilm, which clings to the internal fixation surface. The bacterial biofilm acts as a shield to reduce antibiotic sensitivity and to promote bacterial growth. However, VSD technology can efficiently demolish the bacterial biofilm and raise the rate of bacterial culture that is positive to 71.4%, which is beneficial for the application of antibiotics that are sensitive. Moreover, due to the ongoing suction effect from negative pressure, necrotic tissue and inflammatory substances are removed, thus reducing the absorption of toxic substances in the body and quickly relieving inflammation. In addition, the gradual negative pressure effect on the wound edges helps to improve vascularization of the tissue, thus facilitating the healing process. After reviewing the literature and observing clinical cases, we have found that the negative pressure effect of VSD can generate mechanical force that helps recruit immune cells around the incision edge [31]. This leads to a significant increase in the number and density of neutrophils, which enhances local immune function. It is possible that this is due to the activation and enhancement of cell function by mechanical force [32–35]. However, further research is needed to determine the broader impact of VSD's negative pressure mechanical force on the immune environment and internal stability around the incision edge. In addition, the technical operation of VSD is not very difficult for most spinal surgeons, and it can significantly reduce the number of incision dressing changes, thereby reducing the workload of doctors and nurses. Overall, VSD is a suitable method to consider.

In comparison with traditional negative pressure drainage, VSD technology necessitates continuous negative pressure suction, which is constrained by the length of the negative pressure drainage tube. This limitation results in patients being able to move only around the bedside with a smaller range of motion during treatment, potentially posing a disadvantage of VSD technology [36, 37]. Nevertheless, those with cerebrospinal fluid leakage, poor compliance, and hypersensitivity to VSD dressings after cervical spine surgery with DII should not use VSD technology treatment, as per our experience and literature reports.

## Conclusion

VSD is not recommended for individuals with active cerebrospinal fluid leakage and those who are prone to bleeding, With the limitations on the number of posterior cervical surgeries and the incidence of incision infections, as well as the fact that only a single treatment center case study was conducted, we are presenting the research results as a clinical indication. Nevertheless, we still hope to propose a trial method for the treatment of posterior cervical infections for spinal surgeons to experiment with and choose from.

## Data Availability

All data analysed during this study are included in the manuscript. The datasets used in this article are available from the corresponding author (ZQ.C.) on reasonable request.

## Abbreviations

VSD: Vacuum Sealing Drainage
TND: Traditional Negative Pressure Drainage
DII: Deep Incision Infections
BMI: Body Mass Index
SSI: Surgical Site Infections
ESR: Erythrocyte Sedimentation Rate
CRP: C-reactive Protein
